# Multi-terminal spin valve in a strong Rashba channel exhibiting three resistance states

**DOI:** 10.1038/s41598-018-21760-9

**Published:** 2018-02-21

**Authors:** Joo-hyeon Lee, Hyung-jun Kim, Joonyeon Chang, Suk Hee Han, Hyun Cheol Koo, Shehrin Sayed, Seokmin Hong, Supriyo Datta

**Affiliations:** 1 Center for Spintronics, Korea Institute of Science and Technology, Seoul, 02792 Korea; 2 KU-KIST Graduate School of Converging Science and Technology, Korea University, Seoul, 02481 Korea; 3 School of Electrical and Computer Engineering, Purdue University, West Lafayette, IN 47907 USA

## Abstract

In a strong spin-orbit interaction system, the existence of three resistance states were observed when two ferromagnetic (FM) contacts were used as current terminals while a separate normal metal contact pair was used as voltage terminals. This result is strikingly different from ordinary spin valve or magnetic tunnel junction devices, which have only two resistance states corresponding to parallel (*R*_P_) and antiparallel (*R*_AP_) alignments of the FM contacts. Our experimental results on a quantum well layer with a strong Rashba effect clearly exhibit unequal antiparallel states, i.e., *R*_AP(1)_ > *R*_P_ > *R*_AP(2)_, up to room temperature. The three-states are observed without any degradation when the distance between the non-magnetic voltage probe and the ferromagnetic current probe was increased up to 1.6 mm.

## Introduction

The interplay between spin and charge due to spin-orbit coupling has added a new dimension to spintronics through novel materials such as narrow gap semiconductors^1–3^, heavy metals^4–9^ and topological insulators^10–13^. In conventional magnetoresistive devices e.g. spin valve or magnetic tunnel junction, the resistance state is determined by magnetization alignment of the two ferromagnetic layers and bistable resistance states corresponding to the parallel and antiparallel alignments would exist. In a spin-momentum locking system, the charge current induces a separation of the electrochemical potentials of spin-up and spin-down electrons and subsequently produces a spin voltage. A numerical study^14^ predicted that in a multi-terminal setup with two ferromagnets fabricated on a channel with spin-momentum locking will exhibit three distinct resistance states with unequal anti-parallel resistance. The results of that proposal were explained by a reciprocal relation between direct (conversion of charge to spin) and indirect (conversion of spin to charge) effects, including the observed negative sign, which was derived from the well-established Onsager relation^15,16^. However, three resistance states and a reciprocity of an electrochemical potential measurement have not yet been investigated experimentally in any channel system with spin-momentum locking.

## Results and Discussion

### Electrochemical potential in a strong Rashba system

**Figure 1:**
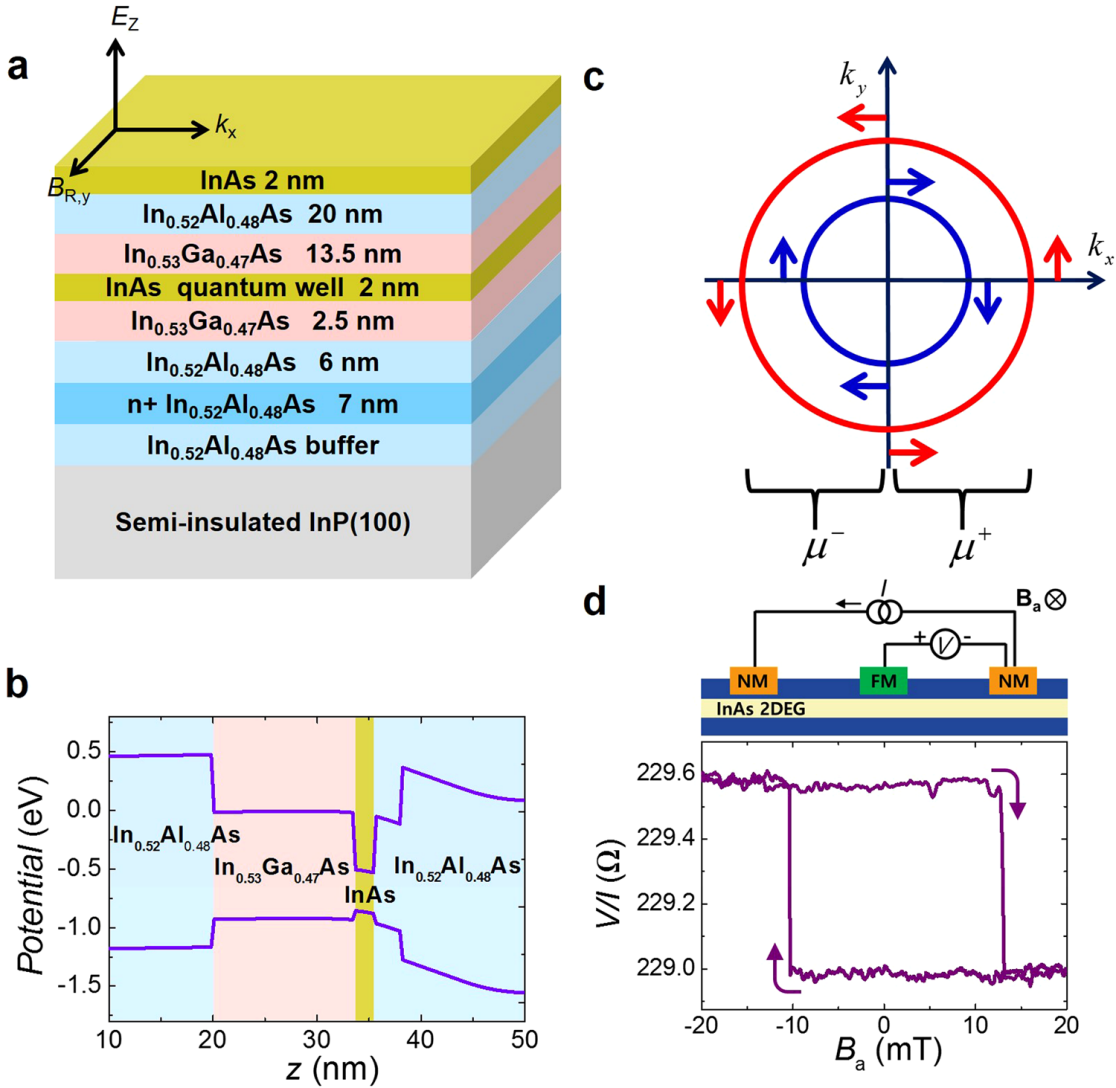
Separation of electrochemical potential in a Rashba system. (**a**) InAs quantum well structure. (**b**) Energy band diagram of the channel structure. (**c**) Fermi circles for a Rashba channel. Positive and negative propagating states are occupied according to chemical potentials μ^+^ and μ^−^, respectively. This structure in turn creates a spin potential (μ^+^ − μ^+^) proportional to the degree of spin polarization of the channel. Red and blue circles correspond to majority and minority spins, respectively. (**d**) Potentiometric geometry and measurement result.

To investigate spin voltage induced by an electrochemical potential shift, an InAs-based heterostructure^2,3^ was utilized as shown in Fig. 1a (see Methods). This system consists of In_0.52_Al_0.48_As/In_0.53_Ga_0.47_As cladding layers and a 2-nm InAs quantum well acting as a two-dimensional electron gas. The In_0.52_Al_0.48_As and In_0.53_Ga_0.47_As cladding layers were determined, according to an energy band calculation (Fig. 1b), to be potential barriers that confined electrons inside the InAs quantum well. An *n*-doped carrier supply layer was inserted only below the InAs active layer, so this asymmetric quantum well produces an intrinsic electric field (*E*_*z*_) and hence moving electrons (*k*_*x*_) that in turn induce the Rashba effective field (*B*_R*y*_) even without a gate electric field^2,3,17^.

As shown in Fig. 1c^18^, the Rashba spin splitting creates a spin potential proportional to the spin polarization of the channel. In the potentiometric geometry^18–22^ shown in Fig. 1d, when the bias current is applied in the quantum well channel, the voltage is measured between the Ni_81_Fe_19_ electrode (FM) and the non-magnetic Ti/Au electrodes (NM) at the end of the channel. The bias current induces a spin potential in the channel i.e. the electrochemical potential split into two values for the up and down spin-polarized electrons. The magnetization direction of the FM is determined by an applied magnetic field. When the magnetization direction of the FM is aligned with the majority (minority) spin direction in the channel, the high (low) spin electrochemical potential of the quantum well channel is measured by the FM contact in the form of a voltage^19,23^. The magnitude of the signal is effectively lowered by the interface spin polarization due to FM which is determined by the majority and minority spin dependent conductances of the FM contact. Note that the detected voltage matches the magnetization direction of the FM and shows a hysteresis loop (Fig. 1d).

### Three resistance states exhibiting charge-to-spin conversion

**Figure 2:**
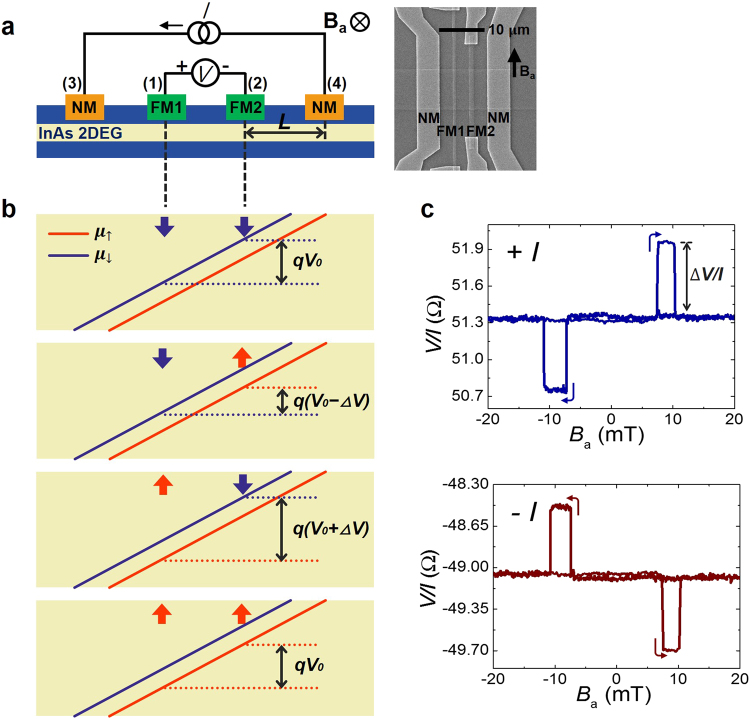
Four-terminal potentiometric measurements exhibiting charge-to-spin conversion. (**a**) Measurement geometry and scanning electron micrograph of device. Centre-to-centre distance *L* = 5.9 μm and *T* = 1.8 K. (**b**) Electrochemical potentials of the channel. The red and blue arrows indicate the directions of electrochemical potentials aligned to the FMs. (**c**) Potentiometric signals for positive and negative bias currents. The applied magnetic field is parallel to the Rashba field. The baseline resistance values for positive and negative currents are not identical because of a semi-tunnelling contact at the metal-semiconductor interface (Supplementary Fig. 3).

For a device with two FMs and two NMs schematically shown in Fig. 2a, the current is applied from one NM to the other NM and the voltage is measured between the FMs (see Methods). The right image of Fig. 2a shows a scanning electron micrograph of the device. This device was made with a channel width of 8 μm and lateral dimensions of the two FMs of 0.6 μm × 40 μm and 1 μm × 25 μm. In this charge-to-spin conversion experiment, the bias charge current creates spin splitting which leads to two separate electrochemical potentials as shown in the potential diagram of Fig. 2b. The voltage detects the difference detected between the two potentiometric FM voltage probes as is shown in Fig. 2c, which clearly shows the three states for different combinations of the magnetization vectors. The magnetizations of the two ferromagnetic electrodes (FMs) have different coercivities because of their different aspect ratios. By sweeping an applied magnetic field in both directions, we observe all four combinations for the magnetization vectors of the two FMs. The baseline voltage representing offset voltage that originates from the ohmic drop depends on the distance between the two FM contacts and the sheet resistance of the quantum well channel.

To explain the mechanism, we assume that the FM magnetized along the spin-up direction is mainly connected to the spin-down electrochemical potential in the channel throughout the paper, and vice versa^24^. As shown in the first panel of Fig. 2b, when the magnetization direction of the two FMs is along the spin-up axis, both voltage probes (FM1 and FM2) read the spin-down electrochemical potential, and the detected voltage is only the ohmic drop (*V*_0_). There is no spin voltage difference in this case. Also, when the magnetization direction of both FMs is along the spin-down direction, the two FM probes read the spin-up potential and the detected voltage difference is also the ohmic drop (*V*_0_) (fourth panel of Fig. 2b). When the magnetization directions of FM1 and FM2 are parallel to the spin-up and spin-down potentials of the channel, respectively, these FMs read the spin-down and spin-up electrochemical potentials, respectively, as shown in the second panel of Fig. 2b. Hence, the voltage probe here shows a lower potential, for which the decrease in the magnitude is due to the electrochemical potential difference between the spin-up and spin-down cases in this channel, and the voltage is *V*_0_ − Δ*V*. Also, when the magnetization direction of FM1 and FM2 are aligned to spin-up and spin-down potentials of the channel, respectively, the voltage probe produces a higher signal, *V*_0_ + Δ*V* (third panel of Fig. 2b). The signals, Δ*V*, in these two cases are identical. When the polarity of the current is reversed, the orientation of the majority spin in the channel is reversed and therefore the signs of Δ*V* is also reversed as shown in Fig. 2c.

### Three resistance states exhibiting spin-to-charge conversion

**Figure 3:**
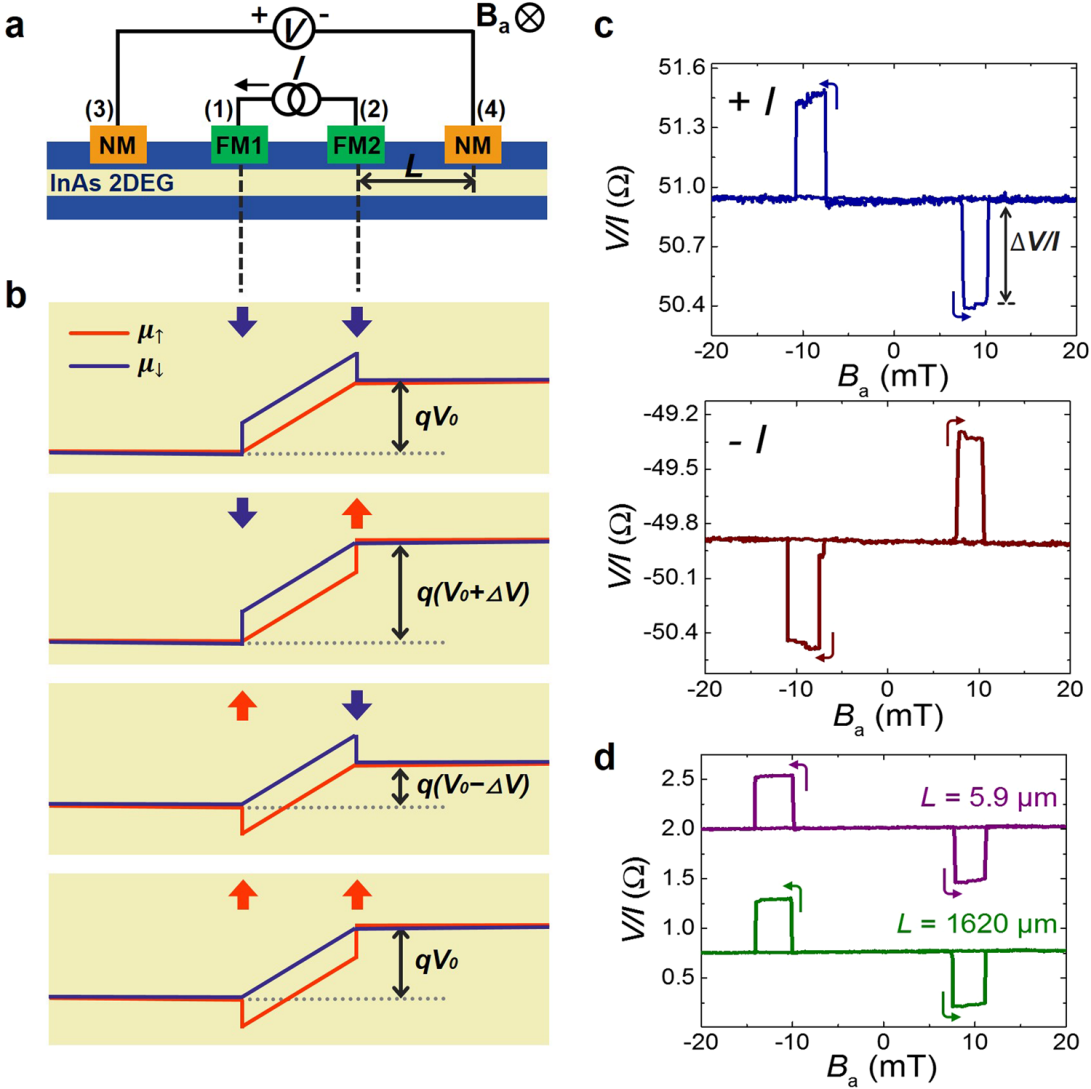
Reciprocal measurements exhibiting spin-to-charge conversion. (**a**) Measurement geometry. (**b**) Electrochemical potentials of the channel. The red and blue arrows indicate the directions of electrochemical potentials aligned to the FMs. (**c**) Reciprocal signals for positive and negative bias currents. *L* = 5.9 μm and *T* = 1.8 K. (**d**) Reciprocal signals for *L* = 5.9 μm and *L* = 1620 μm. Note that the FMs utilized in **c** and **d** have different coercivities.

The reciprocal configuration of the potentiometric geometry is shown in Fig. 3a. To exhibit spin-to-charge conversion, the current is applied from FM1 to FM2 and the voltage is measured between the NMs. In this configuration, there is no current under the non-magnetic electrodes. The diagrams for the spin-up and spin-down electrochemical potentials are illustrated in Fig. 3b. Due to the Rashba spin splitting, two separated electrochemical potentials form where there is current. At the channel regions on the left side of FM1 and the right side of FM2, the spin-up and spin-down electrochemical potentials coalesce into an identical potential because there is no bias current and the Rashba spin splitting does not arise. NM contact measures the average between spin-up and spin-down electrochemical potentials which is the charge potential. Hence, the voltage difference between the NMs will actually measure the relative difference between these flat electrochemical potentials on the regions left of FM1 and right of FM2 respectively.

In the reciprocal measurement, the detected signal is also determined by the magnetization alignment of the two FMs as shown in Fig. 3c. When the magnetization vectors of the two current injectors (FM1 and FM2) is along the spin-up axis, FMs are aligned with spin-down chemical potential in the channel and only the spin-down electrochemical potential changes at the contact. Thus, its value coincided with that of the spin-up electrochemical potential in the region where *I* = 0 as shown in Fig. 3b. A similar mechanism applies for the magnetization of the FM along the spin-down direction as well. As illustrated in the first panel of Fig. 3b, when the two FMs are aligned to spin-down electrochemical potential, the both voltage probes (NMs) read the spin-up electrochemical potential of the channel. Thus, the spin voltage difference is zero, while the ohmic drop appears as a baseline voltage. Also, when both FMs are aligned to the spin-up electrochemical potential of the channel and, again, the detected spin voltage difference is zero (fourth panel of Fig. 3b). When FM1 and FM2 are aligned to the spin-down and spin-up potentials of the channel, respectively, the NMs are aligned to the opposite directions of the aligned potentials, i.e., spin-up and spin-down electrochemical potentials, respectively, as shown in the second panel of Fig. 3b. Hence, the voltage probe shows the higher potential (*V*_0_ + Δ*V*). Using a similar mechanism, when FM1 and FM2 are aligned to spin-up and spin-down potentials of the channel, respectively, the voltage probe shows a lower potential, *V*_0_ − Δ*V* (third panel of Fig. 3b). The signal Δ*V* in the reciprocal geometry is the same as that in the potentiometric geometry shown in Fig. 2, while the two geometries carry the signals of opposite polarity to each other. The polarity of the current also determines the orientation of the majority spin, so the signs of Δ*V* are also determined by the current polarity, as shown in Fig. 3c. In this reciprocal measurement, the distance between FM and NM does not much affect the magnitude of the signal, as shown in Fig. 3d. Surprisingly, the non-magnetic contacts to the channel read the magnetization states of the ferromagnetic electrodes located 1620 μm away from the detector without any signal loss. This observation can be understood from the electrochemical potential view in Fig. 3. Depending on the magnetization direction, FM current injectors are shifting the relative positions of the electrochemical potentials in the regions where *I* = 0 giving rise to the three state signal. However, electrochemical potentials are flat in the region where *I* = 0. Hence, the voltage difference measured by NMs is invariant to the distance between voltage and current probes. The detected signal, Δ*V*, is also a function of the gate voltage, which modulates the strength of the Rashba effect (Supplementary Fig. 1).

### Onsager Reciprocity in a strong spin-orbit interaction system

The Onsager relation is valid for any setup in the linear response regime, even without invoking any spin-related effect. The relation states that the ratio of voltage to current remains the same even when exchanging voltage and current terminals. If time-reversal symmetry (TRS) is broken, then we must reverse all TRS breaking field to satisfy reciprocity. The reciprocity relation for the four-terminal geometry shown in Figs 2 and 3 is given by^14–16^

1 $$\frac{{V}_{12}(+{m}_{1},+{m}_{2})}{{I}_{34}}=\frac{{V}_{34}(-{m}_{1},-{m}_{2})}{{I}_{12}},$$

where the opposite sign of the magnetization direction is necessary to satisfy the reciprocity relation. The detected signals in Figs 2 and 3 are perfectly explained by the Onsager reciprocity relation as described in equation (1). The prediction of unequal anti-parallel states should only be realized in multi-terminal measurements and not two terminal measurements^25^.

**Figure 4:**
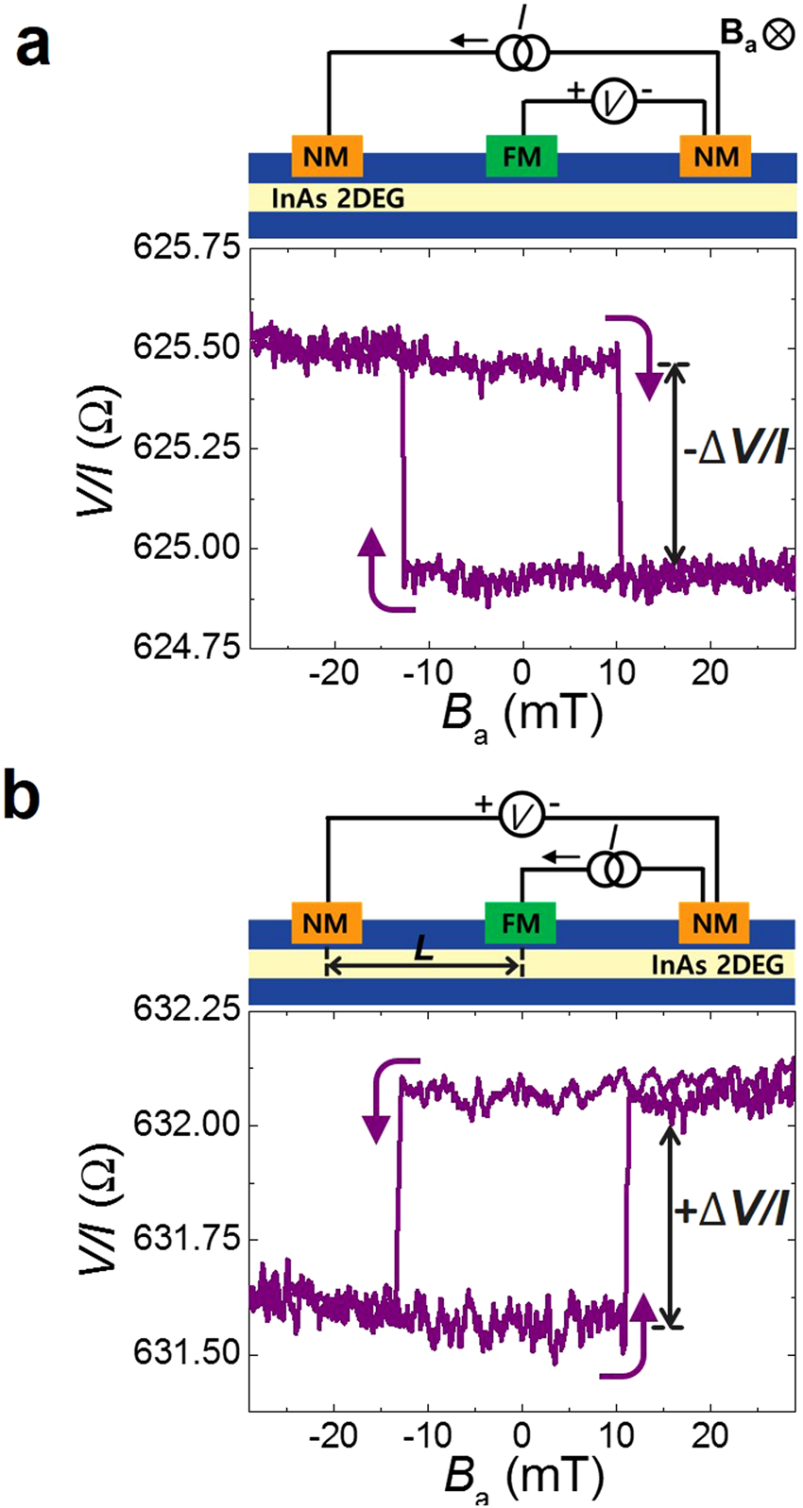
Reciprocity of the three-terminal geometry. (**a**) Potentiometric signal. (**b**) Reciprocal signal. The results in (**a**) and (**b**) show charge-to-spin and spin-to-charge conversion signals, respectively. *L* = 870 μm and *T* = 1.8 K.

The reciprocity relation can be extended to various cases. Another example of using reciprocity for measuring electrochemical potential is shown in Fig. 4. Using one FM and two NMs in a Rashba channel, potentiometric and its reciprocal signals are obtained as illustrated in Fig. 4a and b, respectively. In these two geometries, the spin splitting induced by the charge current is detected by the FM detector and the electrochemical potential alignment induced by the spin current is detected by the NM detectors. The detected potential differences Δ*V* in the two geometries are identical, but with opposite signs of the two signals. This result implies the Onsager reciprocity to be valid for theses diverse cases in the Rashba system.

### Temperature dependence of three-state signal

**Figure 5:**
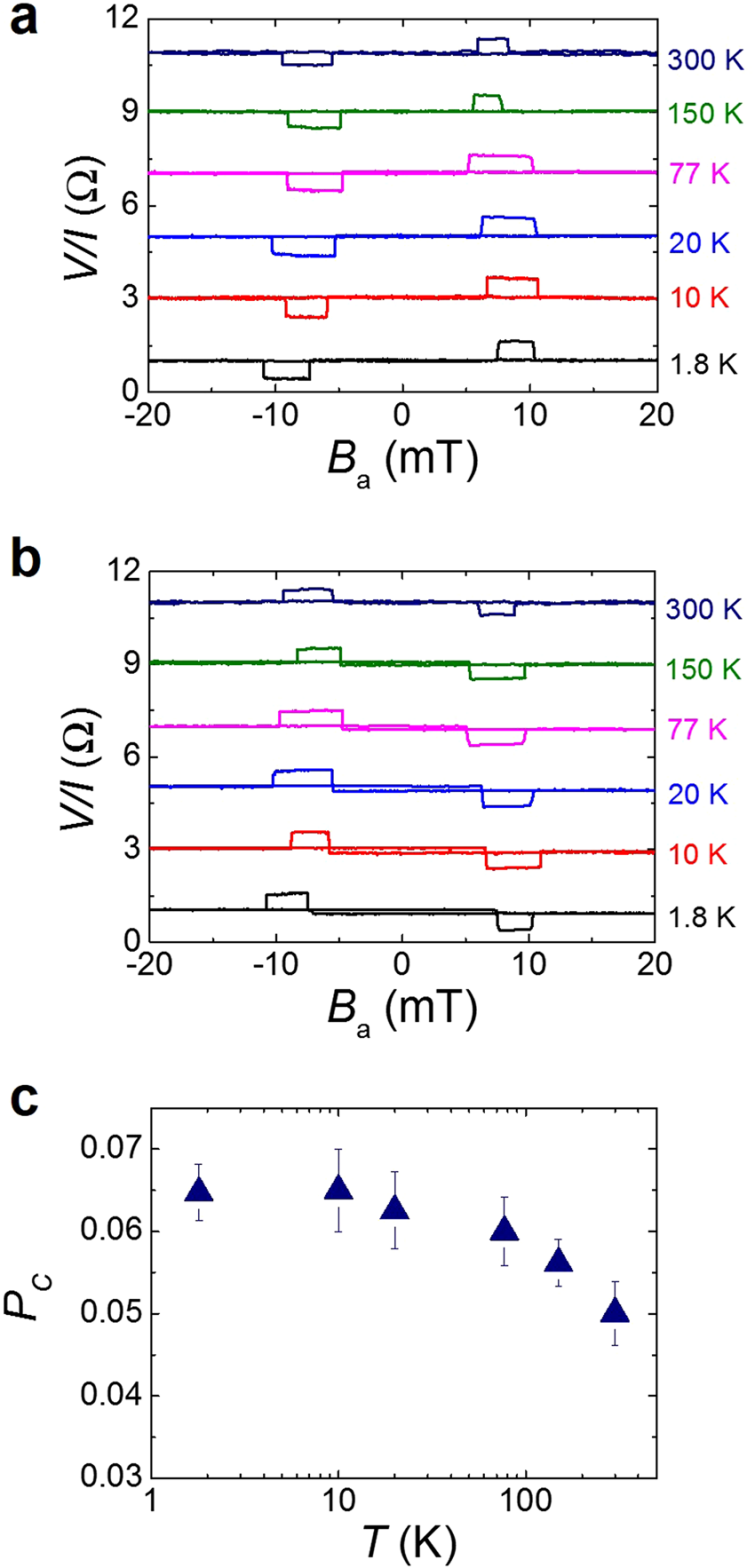
Temperature dependence of reciprocity and spin polarization. (**a**,**b**) Temperature dependence of potentiometric signals (**a**) and reciprocal signals (**b**). Data are offset for clarity. (**c**) Temperature dependence of spin polarization in a channel. *L* = 5.9 μm. Error bars represent the standard deviation.

Figure 5a and b show the temperature dependence of the potentiometric and reciprocal measurements, respectively. Clear signals with reciprocity are observed up to 300 K. From these data, we found that the Rashba field and the separation of the electrochemical potentials occurs up to room temperature. The transition fields slightly change with temperature because the switching field of FM changes with the temperature.

### Estimation of the channel polarization

We consider the experimental data quantitatively. The detected signal from the potentiometric geometry or the reciprocal geometry is given by^14^

2 $${\rm{\Delta }}V=\frac{1}{2}({V}_{12}(+{m}_{1},-{m}_{2})-{V}_{12}(-{m}_{1},+{m}_{2}))=\frac{2{P}_{C}{P}_{FM}\eta }{\pi {G}_{B}}{I}_{34},$$

where *P*_*C*_, *P*_*FM*_, and *G*_*B*_ are the channel polarization, FM polarization, and ballistic conductance of the channel, respectively. The factor η (0 ≤ η ≤ 1) indicates how much current in the channel gets shunted in the FM contact with η = 1 indicating no current shunting.

The precise value of spin polarization in the Rashba channel has not been reported, while the Rashba effective magnetic fields in semiconductors and metals have been estimated^2,6^. Based on the experimental results and equation (2), we estimate the spin polarization of the channel as shown in Fig. 5c. In the four-terminal device, *P*_*FM*_ = 0.5 and η = 1 (Supplementary Fig. 2) are assumed. Also, we use the ballistic conductance, *G*_*B*_, which is independently measured to be 34.9 mA/V. The extracted spin polarizations of the channel are 0.065 at 1.8 K and 0.05 at 300 K. While the channel polarization slightly decreases with increasing temperature due to the thermal agitation at a higher temperature, a clear spin polarization is still detected at room temperature. The channel polarization is relatively constant up to 77 K and shows a small decrease (~20%) at room temperature. This temperature degradation of the signal is slightly larger than a typical InAs quantum well channel (~10%)^26,27^. The temperature dependence of Rashba effect has not been clearly understood yet. We believe that the reason for degradation of Rashba effect at higher temperature is due to a severe mobility decrease of the channel, suggesting the thermal fluctuation and averaging effects due to scattering events.

## Summary

We observed the separation of electrochemical potentials for spin-up and spin-down electrons. Using multi-terminal spin valve geometry, long range signals of three distinct resistance states with reciprocity were observed up to room temperature. We expect our results to be found for any materials exhibiting a spin-momentum locking phenomenon and our methodology to be applied to diverse spin information devices.

## Methods

### Device Fabrication

A two-dimensional electron channel was epitaxially grown using a molecular beam epitaxy system. The vertical structure from top to bottom is InAs (2 nm), In_0.52_Al_0.48_As (20 nm), In_0.53_Ga_0.47_As (13.5 nm), InAs active layer (2 nm), In_0.53_Ga_0.47_As (2.5 nm), In_0.52_Al_0.48_As (6 nm), *n*^+^ In_0.52_Al_0.48_As carrier supplier (7 nm), In_0.52_Al_0.48_As buffer (300 nm), and a semi-insulating InP (001) substrate. The In_0.52_Al_0.48_As and In_0.53_Ga_0.47_As double cladding layers were the potential barrier to confine the electrons in the quantum well. The channel was fabricated using photolithography and Ar ion milling. The patterns for ferromagnetic (Ni_81_Fe_19_) and non-magnetic (Ti/Au) electrodes were made by electron beam lithography. The thicknesses of Ni_81_Fe_19_ and Ti/Au electrodes are 80 nm and 10 nm/100 nm, respectively. Parts of the semiconductor layers were milled out to adjust the interfacial resistance, and therefore the distance from the ferromagnetic electrode to the quantum well layer is 23 ± 2 nm.

### Measurements method

A vibrating sample magnetometer was used for acquiring magnetization curves of thin film. Anisotropic magnetoresistance was measured for investigating the switching fields of ferromagnetic electrodes (Supplementary Fig. 4). Potentiometric and its reciprocal measurements were taken inside a temperature controlled cryostat for probing the electrical characteristics. We utilized Keithley 236 as a current source and Keithley 2182 A as a voltmeter. The impedance of voltage meter is 10 GΩ.

### Data availability

The data that support the findings of this study are available from the corresponding authors on request.

## Electronic supplementary material

Supplementary Information
