# Supplementary material for: Multi-terminal spin valve in a strong Rashba channel exhibiting three resistance states
Source: Sci Rep. 2018 Feb 21;8:3397. doi: 10.1038/s41598-018-21760-9 (PMC5821859; doi:10.1038/s41598-018-21760-9)
Supplement: Supplementary file 1 — Supplementary Information [file 41598_2018_21760_MOESM1_ESM.pdf]

Supplementary Information for "Multi-terminal spin valve in a strong Rashba channel exhibiting three resistance states"

Joo-hyeon Lee[1,2], Hyung-jun Kim[1], Joonyeon Chang[1], Suk Hee Han[1], Hyun Cheol Koo[1,2]*, Shehrin Sayed[3], Seokmin Hong[1,3] and Supriyo Datta[3]

[1]Center for Spintronics, Korea Institute of Science and Technology, Seoul 02792, Korea

[2]KU-KIST Graduate School of Converging Science and Technology, Korea University, Seoul, 02481, Korea

[3]School of Electrical and Computer Engineering, Purdue University, West Lafayette, IN 47907, USA.

Correspondence and requests for materials should be addressed to H.C.K. (email: hckoo@kist.re.kr)

Contents

Supplementary Fig. 1. Gate control of electrochemical potential separation.

Supplementary Fig. 2. Bias current shunting.

Supplementary Fig. 3. I-V curves at ferromagnet-quantum well junction.

Supplementary Fig. 4. Magnetic behaviour of ferromagnetic film and patterns.

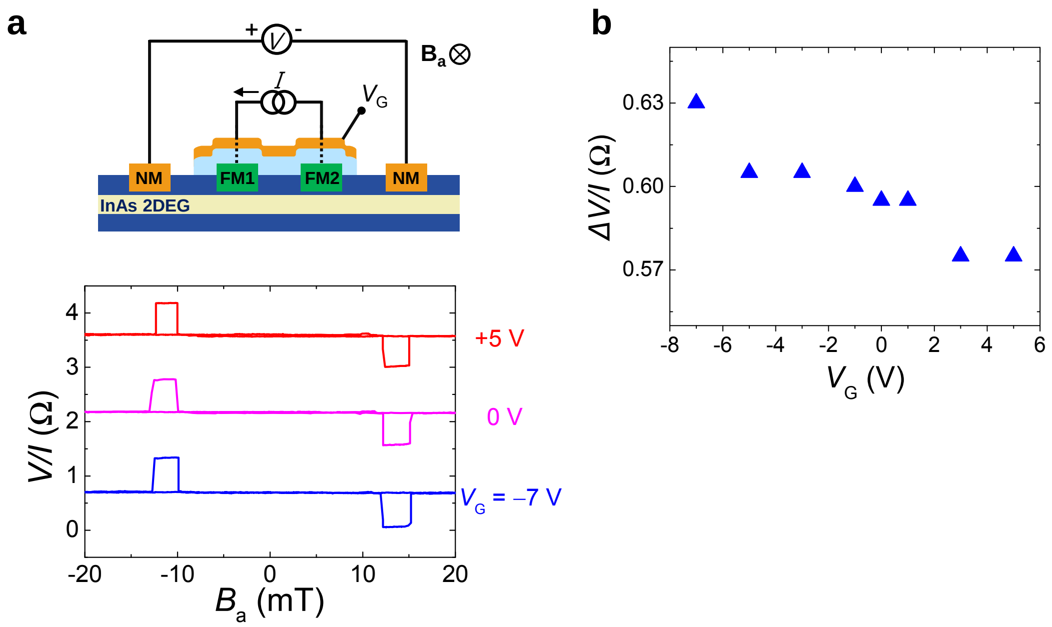

Supplementary Fig. 1. | Gate control of electrochemical potential separation. (a) Measurement geometry and results of reciprocal measurement for various gate voltages. Data are offset for clarity. (b) Gate voltage dependence of the reciprocal signal. The Rashba effect decreases with increasing gate voltage in our channel structure. The signal in the reciprocal measurement shows the same trend as the Rashba parameter. The thickness of the SiO_2 gate oxide is 115 nm.

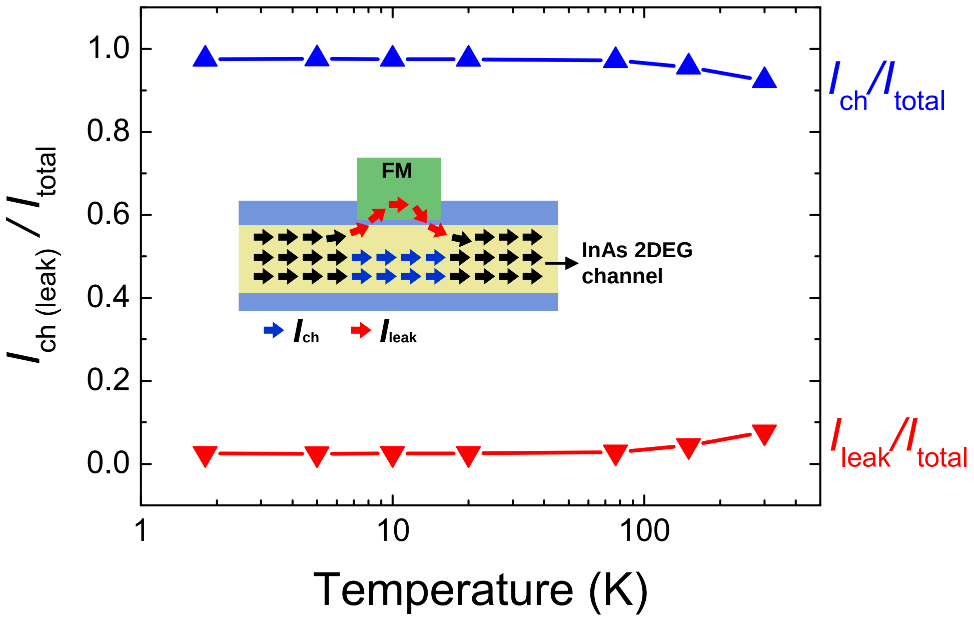

Supplementary Fig. 2. | Bias current shunting. Below the ferromagnetic electrode, the total current, I_{total}, is divided into I_{ch} and I_{leak}. Electrochemical potential signals are noticeably reduced if the leakage current (I_{leak}) into the ferromagnetic electrode is high. In our case, the leakage current is negligible and $\eta \approx 1$ for the entire temperature range. The factor η is how much current in the channel gets shunted in the FM contact. $\eta = 1$ indicates no current shunting.

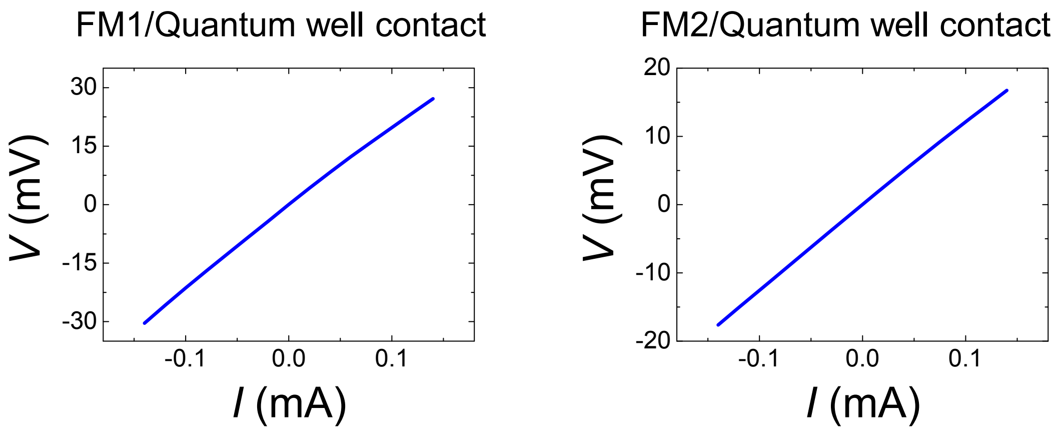

Supplementary Fig. 3. | I-V curves at ferromagnet-quantum well junction. The contact areas for FM1 and FM2 are 0.6 μm × 8 μm and 1 μm × 8 μm, respectively.

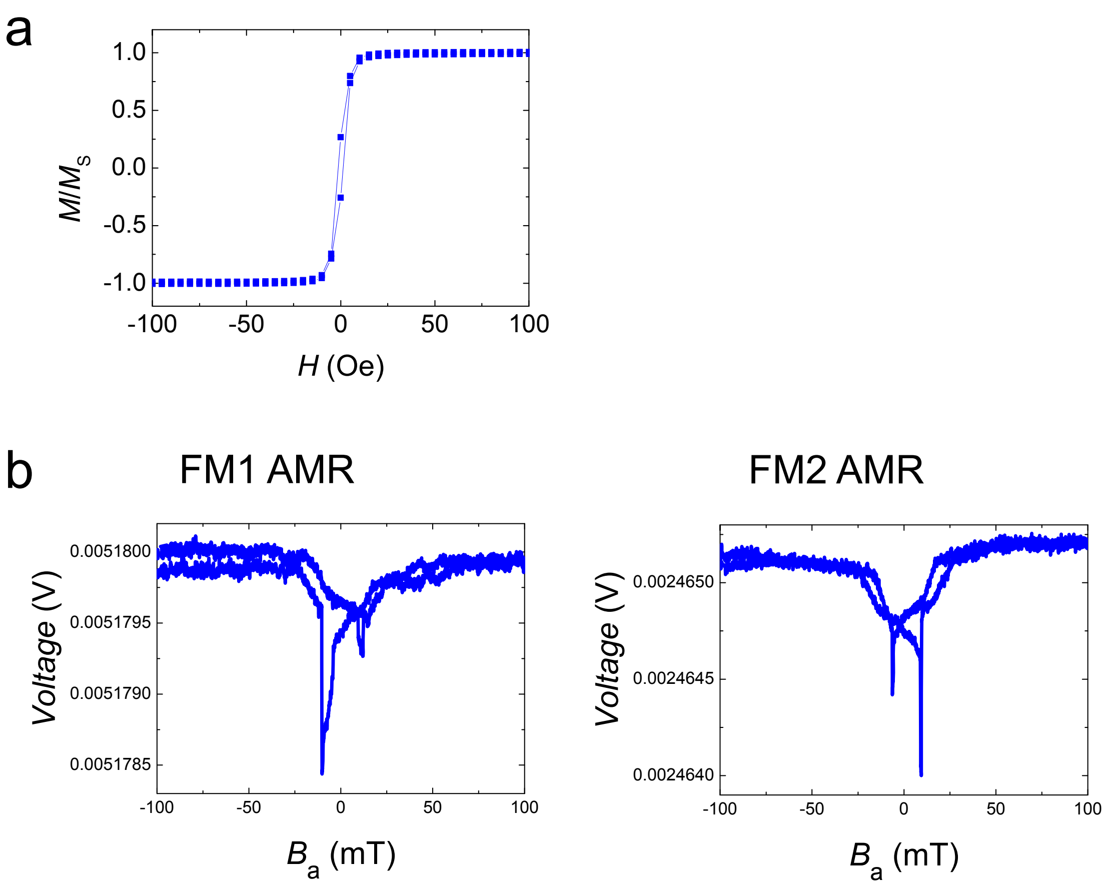

Supplementary Fig. 4. | Magnetic behaviour of ferromagnetic film and patterns. Results of (a) vibrating sample magnetometer and (b) anisotropic magnetoresistance measurements. In anisotropic magnetoresistance measurements, the lateral dimensions of two FMs are 0.6 μm × 40 μm and 1 μm × 25 μm, respectively. The dip points correspond to the switching fields of FM patterns.
